# A Rare Case of Vaginal Gastrointestinal Stromal Tumor (GIST) Initially Perceived as a Polypoid Lesion

**DOI:** 10.7759/cureus.70947

**Published:** 2024-10-06

**Authors:** Theodoros Mariolis-Sapsakos, Eirini Nannou, Vassilis Milionis, Stavros Angelis, Dimitrios Filippou

**Affiliations:** 1 Anatomy, National and Kapodistrian University of Athens, Athens, GRC

**Keywords:** extra gastrointestinal gist, gastrointestinal stromal tumour, gist, imatinib, vaginal, vulvar gist

## Abstract

Gastrointestinal stromal tumors or GISTs are the most common mesenchymal tumors of the gastrointestinal tract; only a few cases have been reported at other sites. The prognosis of GISTs depends on their size and mitotic rate. Surgical removal is the treatment of choice although imatinib shows clinical benefits.

We present a rare case of a GIST in a 64-year-old female patient treated for vaginal bleeding. During diagnostic hysteroscopy, an intra-uterus polyp and a large vaginal mass were identified and multiple biopsies were obtained. Initially, the histological examination of the first lesion suggested that it was an intra-uterus, benign adenomatous polyp but later the second lesion indicated a malignant vaginal neoplasm. The patient was operated on and the postoperative course was uneventful. The histological analysis of the specimen finally revealed a GIST.

Vaginal GISTs are extremely rare tumors and in most cases, diagnosis is achieved only histologically in the removed specimen. Additional treatment options and prognosis are similar to those of gastric GISTs.

## Introduction

Gastrointestinal stromal tumors or GISTs are the most prevalent mesenchymal malignancies of the gastrointestinal tract [1]. Roughly 70% arise in the stomach, 20-30% in the small intestine, and less than 10% in the esophagus, colon, and rectum [1]. However, a small number of GISTs are reported to stem from extra-gastrointestinal masses such as in the omentum, mesenteries, retroperitoneum, rectovaginal septum, and other sites [2]. It is widely known that GISTs have gain-of-function mutations of the c-kit receptor tyrosine kinase gene, which ends in triggering a tyrosine kinase receptor encoded by the proto-oncogene c-kit (KIT, also named CD117 or stem cell factor receptor) [2]. The outcome is the activation of cell multiplication and apoptosis repression [2]. Usually, transformations are concealed in the platelet-derived growth factor receptor alpha (PDGFRA) gene or the c-kit gene [2].

The prognosis of GISTs, either gastrointestinal or extra-gastrointestinal, depends on their size and mitotic rate [3]. GISTs are further grouped into four categories according to their size and mitotic rate: very low, low, intermediate, and high risk [3]. Complete excision should be the cornerstone of GIST treatment [4]. Imatinib, a tyrosine kinase inhibitor, has been shown to be effective in treating GISTs [4]. The ATP-binding pocket is blocked by this inhibitor, leading to the cessation of phosphorylation and activation of the KIT and/or PDGFRA gene [4]. As a consequence, if the tumor is inoperable, adjuvant therapy with imatinib treatment is recommended [4]. Imatinib is associated with mild to moderate adverse effects that include nausea, diarrhea, myalgia, and edema [5]. Around 31-44% of patients on imatinib treatment may experience cutaneous reactions [5].

Primary extragastrointestinal GISTs (EGISTs) of vaginal wall origin are extremely rare. In this case report, we describe a case of vaginal EGIST and discuss its pathogenesis according to available literature.

## Case presentation

A 64-year-old female patient proceeded to complain of vaginal bleeding that started in the past 10 days. The patient’s history revealed no other existing pathologies or chronic diseases. Physical examination was in order and the required blood tests were within normal values. A diagnostic hysteroscopy was performed under local anesthesia. During the hysteroscopy, an intra-uterus polyp and a large vaginal mass were identified and multiple biopsies were obtained. The histological examination of the first lesion suggested that it is an intra-uterus benign adenomatous polyp. Histological analysis of the second lesion suggested a malignant vaginal neoplasm with a maximum diameter of 1.8 cm. It was histologically characterized by intertwining bundles of spindle-shaped and/or oval cells with mild nuclear atypia, eosinophilic cytoplasm, and indistinct cell borders growing in a fibrovascular substrate.

**Figure 1:**
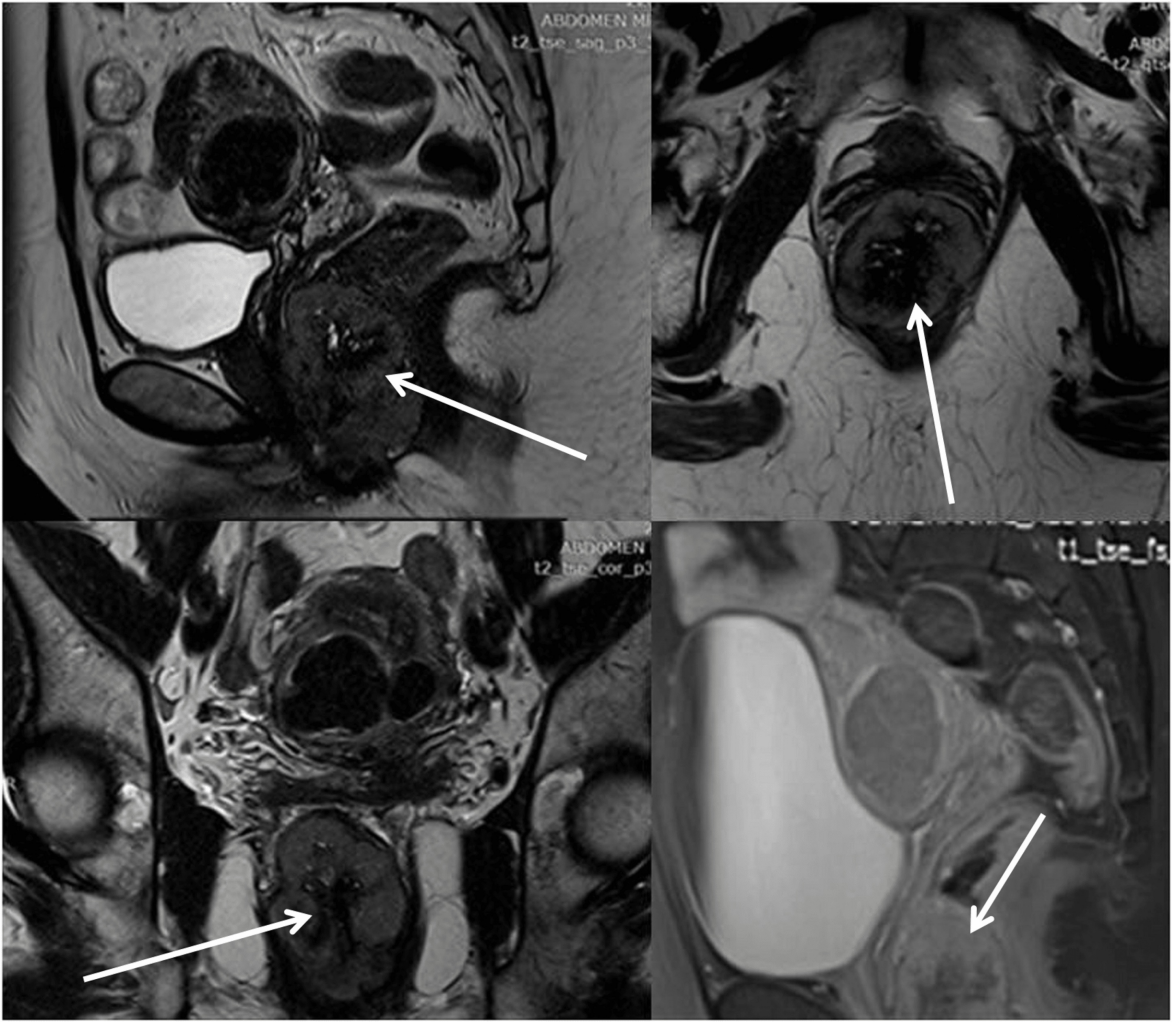
Abdominal and pelvic MRI revealed a sizeable compact lesion (arrows) between the dorsal area of the vagina, cervix, and rectum (5.9 cm craniocaudal, 4.2 cm transverse, and 4.2 cm anteroposterior diameter) It demonstrates moderate hypervascularity, especially at its intensively compact portion and it seems to compress the nearby intestines. MRI, magnetic resonance imaging

The abdominal and pelvic MRI performed revealed a sizeable compact lesion found between the dorsal area of the vagina- cervix and rectum (5.9 cm craniocaudal, 4.2 cm transverse, and 4.2 cm anteroposterior diameter). It descends to the perineum base at the vulva region on the left side of the midline. It demonstrates moderate hypervascularity, especially at its intensively compact portion, and it seems to compress the nearby intestines. There was no evidence of dilatation of the uterine cavity (Figure 1).

**Figure 2:**
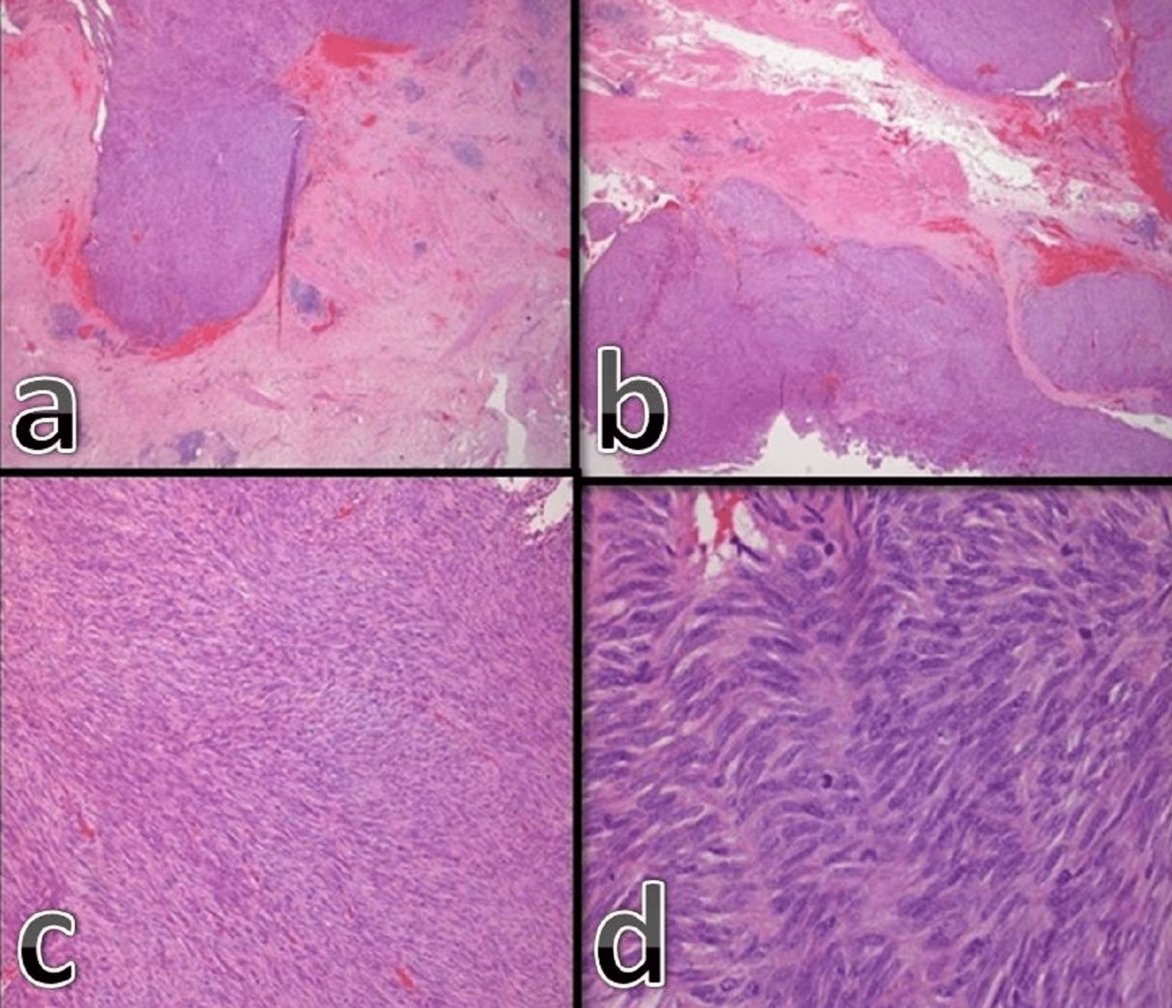
Hematoxylin-eosin stain (a), of a solid tumor invading vaginal wall (X25) (b), in a vaguely fascicular arrangement of neoplastic spindle cells (x100) (c), with elongated nuclei, inconspicuous nucleoli and sparse mitoses (x400) (d)

**Figure 3:**
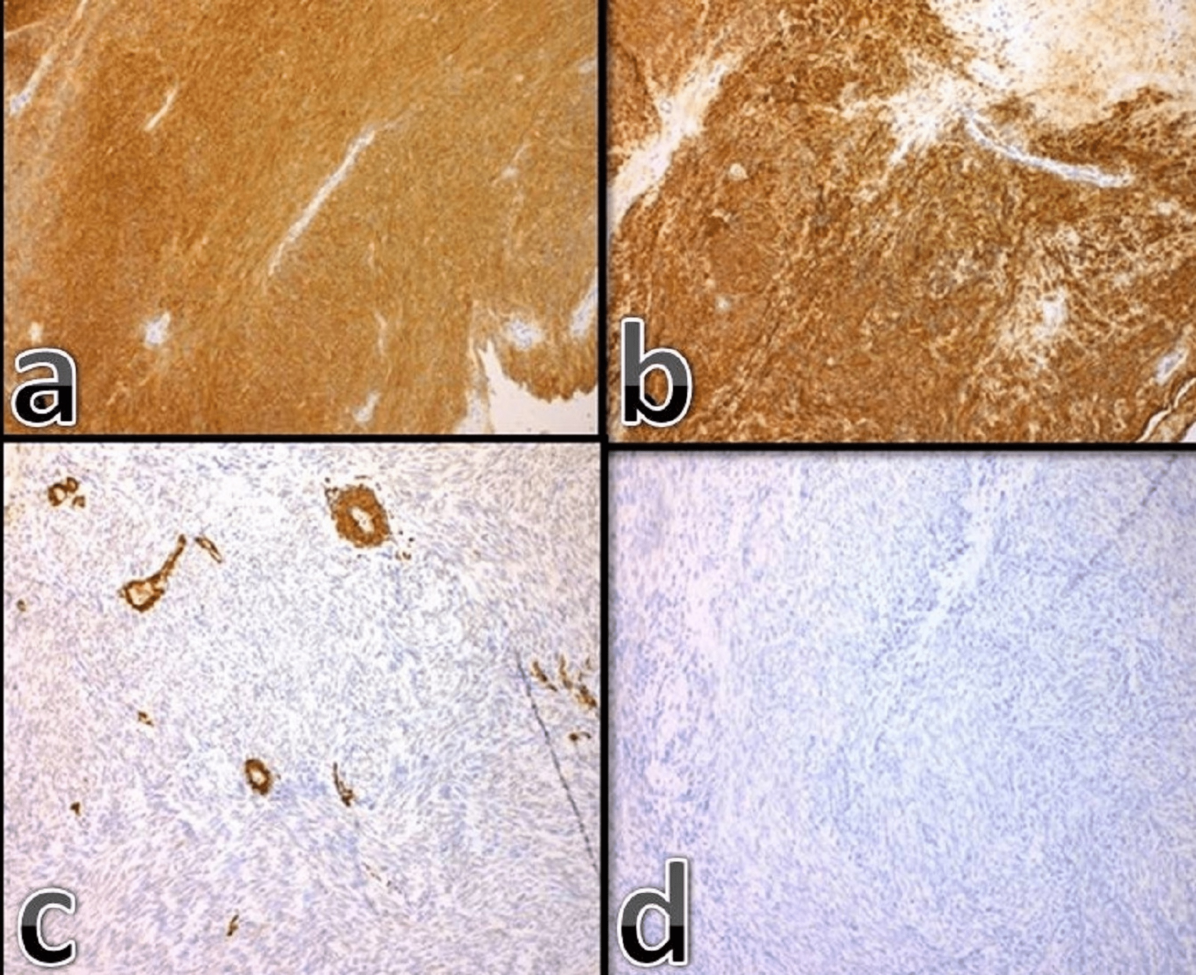
Strong and diffuse immunoreactivity of the tumor cells (a) for CD117 (X100) and (b) CD34 (x100) Negative staining pattern (c) of SMA (x100) and (d) SOX-10 (x100)

The patient was operated on. The mass, which was invading the vaginal wall, was removed oncologically in clear margins and sent for biopsy. The histological examination of the specimen revealed a solid mesenchymal tumor with special characteristics, indicating a stromal mesenchymal GIST. The hematoxylin-eosin stain of the tumor showed the invasion of the vaginal wall, the vaguely fascicular arrangement of neoplastic spindle cells, and the presence of elongated nuclei, inconspicuous nucleoli, and sparse mitoses (Figure 2). Furthermore, the tumor cells presented strong and diffuse immunoreactivity for CD117 and CD34 and negative staining patterns for SMA and SOX-10 (Figure 3).

The postoperative course of the patient was uneventful, and the patient was discharged two days later. The patient did not receive any other treatment and six months later is free of disease with no clinical or imaging evidence for local recurrence or metastatic disease.

## Discussion

Extra-gastrointestinal GISTs are very rare and may arise in abdominal or non-abdominal areas. There are few reported cases of primary vaginal GISTs, and it has been proposed that they may stem from the rectovaginal septum [6-8].

The identification of GIST is based on external morphological features, genetic characteristics on a molecular level, and specific immunophenotypes. When cross-sectioned, GISTs commonly appear as nodular hemorrhagic solid masses [9]. GISTs consist of three types: spindle cell in 70% of the cases, epithelioid (20%), and mixed (10%). The spindle type is similar to leiomyoma [9]. Furthermore, a novel classification system was developed by the National Institute of Health. According to this terminology, GIST can be very low to high danger, and it is based on tumor anatomical position, size, and number of mitoses. Generally, high-risk tumors are usually larger than 5 cm with more than 5 mitoses per 50 HPFs [9]. According to Hou et al., most EGISTs with abdominal or retroperitoneum origins are malignant or at least borderline [10].

A review of the literature revealed five cases EGISTs of the vagina [11-16]. All females suffering were postmenopausal and the tumor was diagnosed clinically as a large growth protracted from the vaginal opening (introitus). The mean tumor size was estimated at 4.5 cm (ranging from 2 cm to 8 cm). Vaginal EGISTs usually stem from the rear wall of the vagina. All reported cases of EGISTs originated from the rectovaginal septum suggesting that these tumors either emanate from the rectovaginal septum or may spread from the rectum [11-16].

In our case, pathological findings were certainly identical to all reported cases. All tumors were well-defined growths that were initially misdiagnosed as leiomyomas [8,11-16]. Typical spindle cell pathology was reported on all occasions [8,11-16]. CD117 was defined in all the reported cases, as expected in all GISTs [8,11-16]. S100, desmin, and SMA (smooth muscle actin) were not defined in any case, so the diagnosis of smooth muscle tumors and melanomas was excluded. In only two out of five cases was molecular profiling conducted [8,11-16]. The Exon 11 KIT mutation was revealed in these cases. A mitotic rate varying between 1 and 25/50HPF was reported [8,11-16]. No vaginal GIST was metastatic [11-16]. It is suggested that these tumors have sluggish behavior. Only one of the five patients was not surgically treated [11-16]. The inoperable tumor was treated with adjuvant therapy with imatinib [8,11]. Furthermore, out of the four cases that were followed up, only one was documented with local recurrence and was thereupon treated with repeated removal and adjuvant imatinib [8,11]. All four cases fully responded and no local or distant recurrence was reported [8,11-16].

## Conclusions

Gastrointestinal stromal tumors (GISTs) are the most common mesenchymal tumors of the gastrointestinal tract. Vaginal GISTs are extremely rare tumors and in most cases, diagnosis is achieved only postoperatively by the histological examination of the removed specimen. Early diagnosis may ensure an acceptable therapeutic result. Appropriate surgical resection is the treatment of choice while additional treatment options and prognosis are similar to gastric GISTs.
